# mTOR Substrate Phosphorylation in Growth Control: An Update

**DOI:** 10.3390/cancers18121944

**Published:** 2026-06-15

**Authors:** Don Benjamin, Michael N. Hall

**Affiliations:** Biozentrum, University of Basel, Spitalstrasse 41, 4056 Basel, Switzerland

**Keywords:** mTOR, cancer, cell signaling, phosphorylation, metabolism, autophagy

## Abstract

**Background:** The mechanistic target of rapamycin (mTOR) is a highly conserved serine/threonine protein kinase that integrates inputs on nutrient status, energy levels, and growth factor stimulation to accordingly regulate cell growth and metabolism. It does this by activating or repressing target proteins covering a broad array of cellular processes. mTOR nucleates two structurally and functionally distinct protein complexes, mTORC1 and mTORC2. Because of their wide-ranging effects in the cell, both mTOR complexes are presumed to have a large number of targets. However, only a relatively small number have been conclusively identified. **Methods:** With emphasis on mammalian mTOR, we previously reviewed the extensive mTOR literature (1991–2021) and compiled a list of all reported substrates of mTORC1 and mTORC2. We have updated this list for the period 2022–2025. **Results/Conclusions:** Many of the targets are involved in autophagy, underscoring the major role of mTOR in the regulation of this process. From the perspective of this Special Issue, targets linked to cancer may be responsible for executing an mTOR-driven pro-oncogenic program and merit future study.

## Simple Summary

mTOR is a central signaling hub that senses diverse inputs relating to cellular energy levels and nutrient status to coordinate cell metabolism and growth appropriately based on these input levels. The wide range of processes controlled by mTOR presupposes a befittingly large number of target proteins. Nevertheless, the actual number of known mTOR targets remains modest, although this number is gradually increasing. We previously listed all direct mTOR targets that had been reported up to 2021. This review updates the list through 2025. Among the new targets are several implicated in cancer and autophagy.

## 1. Introduction

TOR (Target of Rapamycin) is a master regulator of cell growth and metabolism. It senses a wide range of inputs relating to nutrient status, energy levels and growth factors [1]. In response to these various inputs, TOR phosphorylates its targets to execute a metabolic program that befits the level of the inputs. TOR is therefore a critical mediator of homeostasis to ensure balanced cell growth. The importance of TOR can be gauged by the fact that it is essential in all eukaryotes.

Under favorable conditions, TOR (henceforth referred to as mTOR for the mammalian version that is the main focus of this review) promotes an anabolic program that increases cell mass [2] by positively regulating ribosome biogenesis and the synthesis of cellular components such as proteins, nucleotides and lipids. As these are prerequisites for cell growth and division, mTOR is frequently overactivated in cancers or hyperproliferative disorders [3]. mTOR inhibitors of the rapamycin class are clinically approved for some types of cancers and there has been considerable effort to develop second-generation drugs that directly inhibit mTOR kinase activity [4,5].

Conversely, under nutrient-poor or low-energy conditions, mTOR signaling is reduced, and the cell switches to a self-preservation program by activating catabolic processes that restore depleted energy stores or recycle resources in anticipation of more favorable future conditions [6]. This is chiefly effected by lifting the suppression of autophagy that is usually kept quiescent by mTOR signaling. Autophagy (“self-eating”) is the cellular recycling system that degrades old, damaged or dysfunctional organelles or proteins and releases their building blocks for further use in the cell. Basal autophagy is essential as a clearing mechanism to maintain cellular homeostasis. Autophagy, however, is selectively induced by mTORC1 inhibition during starvation as a survival mechanism by releasing energy and resources through the cannibalization of cellular components that are no longer required when cells are in “lock-down” mode. Due to their rapid increase in size and cell numbers, past a certain size, tumors are chronically short of energy and nutrients mainly due to the rapid consumption and depletion of available resources, and to poor vascularization in solid tumors that prevents an adequate supply of oxygen and nutrients, particularly in the tumor center. Thus, in advanced tumors, autophagy supports growth by nutrient re-cycling and can even be cytoprotective during chemotherapy. In this context, while autophagy can be viewed as beneficial for the tumor, this may be a somewhat simplistic view. While autophagy indeed enables an established tumor to cope with nutrient scarcity, there is evidence that preventing it during early stages of cancer delays disease progression. This has led to it being described as a double-edged sword in cancer therapy [7,8], making anti-cancer treatment with mTOR inhibitors of questionable benefit due to the ambivalent role of autophagy in a particular tumor. Depending on the stage, tissue type, underlying mutation, or other specific property of a particular tumor, mTOR inhibition may or may not provide clinical benefit due to the activation of autophagy. This paradoxical situation may be due to the relative importance of mTOR signaling vs. autophagy at a particular stage in tumor progression and has been proposed as a potential reason for the mixed performance of mTOR-inhibiting rapalogs as anti-cancer agents. Identification of tumor biomarkers for mTOR signaling and autophagy may allow patient stratification and individualized therapy of patients who respond to treatment. In addition, therapeutic strategies are under investigation combining mTOR inhibitors with autophagy-inhibiting drugs such as chloroquine with the rationale that inhibiting mTOR signaling while simultaneously preventing the compensatory activation of autophagy can lead to cellular collapse [9,10] and possible clinical benefit [11].

## 2. mTOR Complexes

mTOR is an atypical serine/threonine kinase. It is, however, never found in isolation but as the core catalytic sub-unit of a multi-protein complex. mTOR nucleates two structurally and functionally distinct complexes—mTOR complex 1 (mTORC1) and mTORC2. mTORC1 consists of mTOR, mLST8 and the defining sub-unit Raptor, whilst mTORC2 consists of mTOR, mLST8 and the defining sub-units Rictor and mSin1. Both complexes assemble into homodimers for their active form. Despite sharing the same catalytic unit, the two complexes phosphorylate completely distinct sets of targets. This difference indicates that target recruitment is not performed by mTOR itself in most cases (ZNRF2 may be a rare exception that binds directly to the mTOR sub-unit of mTORC1 for subsequent phosphorylation [12]), but by its associated proteins. Indeed, there is very little sequence similarity between identified mTOR target sites beyond a preference for a proline residue immediately following a target serine or threonine [13]. This suggests that mTOR is a promiscuous kinase with little to no sequence requirement adjacent to the phosphorylated serine or threonine. Moreover, both complexes regulate different cellular processes [14]. mTORC1 is responsive to nutrient availability and energy levels, positively regulates protein translation, cellular metabolism, biosynthesis and is a negative regulator of autophagy. mTORC2 is induced mainly by growth factors and influences cytoskeletal dynamics as well as issuing pro-survival signals. In reality, there is overlap in the processes mediated by the targets of the two complexes, indicating that the division of functions of the two complexes is not strict.

## 3. mTOR and Cancer

The importance of mTOR signaling in cancer biology is demonstrated by the presence of tumor suppressors and oncogenes in its signaling network [15]. Two of the most common tumor suppressors mutated in cancers, TSC and PTEN, are upstream negative regulators of mTORC1 and mTORC2 and their loss of function results in constitutive mTOR activity. LKB1 also serves as a negative regulator of mTOR via AMPK and has a tumor suppressive role, as its mutation results in Peutz-Jeghers syndrome and various cancers. On the other hand, downstream targets of mTOR include commonly activated oncogenes. The mTORC2 target Akt is a major oncogene that is hyperactivated in many different types of cancers. mTORC2 is the activating kinase for the AGC family of kinases (of which Akt is a member) that includes PKA, the PKCs, SGK and PKG, which are involved in tumorigenesis, tumor progression and metastasis. Surprisingly, very few activating mTOR mutations have been reported, indicating that mTOR activation itself only rarely drives tumorigenesis. Rather, it appears that oncogenic mutations commandeer mTOR’s normal physiological role in promoting cell growth and metabolism to support unrestricted cell proliferation, leading to the prevalence of constitutively active mTOR signaling in cancer.

## 4. mTOR Targets

The publication of our review on mTOR substrates in 2022 was positively received by the mTOR community as an important resource [13]. The rapid pace of mTOR research generates an ever-increasing number of publications added to what is already a large body of literature (62,132 results for mTOR in PubMed, as of March 2026), and the compilation of all experimentally verified mTOR targets in a single review was regarded as a valuable contribution to the field.

Since then, we have monitored the literature and now provide an updated list of substrates. We apply the same inclusion criteria as previously applied, namely limiting to mammalian mTOR substrates that are experimentally verified by in vitro kinase phosphorylation, and with the identification of the target site. As before, datasets from global phosphoproteomic studies based on the correlation of phosphorylation status with mTOR inhibition or activation were omitted, as such sites may be phosphorylated downstream of mTOR and thus require further validation as direct mTOR targets.

### 4.1. mTORC1 Targets

We searched PubMed for the following terms: mTOR, mTORC1 and mTORC2 for the period (1 Jan 2022–31 December 2025) and curated the literature. There were 14 novel mTORC1 substrates bearing 29 phosphosites (Table 1). Around half (14) are S/T-P sites, which is a preferred motif for mTOR phosphorylation. There was a strong bias for serine (22 sites) over threonine (7 sites) despite the roughly equivalent abundance of these residues in the human proteome (~8% and ~6% respectively). These observations have long been recognized, and these additional examples support, but do not add to, the known characteristics of mTORC1 phosphorylation.

**Table 1:** Direct substrates of mTORC1.

Substrate	Uniprot ID	Phosphorylated Site(s)	Phosphorylated Site(s) and Adjacent Residues	TOS Motif	Purpose of Phosphorylation	Function	Reference
AMPKb1	Q9Y478	S182	SELSSSPPGPY		Unknown		[16]
AMPKb2	O43741	S184	RDLSSSPPGPY		Inhibitory	Proliferation, nuclear AMPK	[16]
ATF4	P18848	S59,S69 S166,T173 S248	AKAGSSEWLAVDGLVSPSNNS QPLPLSPGVLSSTPDHSF NRSLPSPGVLC	FDLDA	Activating	Mitochondrial stress response	[17]
CDH1	Q9UM11	T129	KKGLFTYSLST		Complex unbinding	APC/C inactivation	[18]
GCN2	Q9K2P8	S230	ILHGGSPDFVGN		Activating	Starvation response	[19]
HOXB13 ^1^	Q92826	T8 S31 T41	PGNYATLDGAK NLVAHSPLTSH HPAAPTLMPAV		Activating	Transcriptional activation Interaction with SKP2	[20]
LST2	Q9HCC9	S670	ELHAGSPSAHE	FFMDD	Stabilizing	Promotes LST2 ubiquitination	[21]
MFSD12	Q6NUT3	T254	EPGEHTPLLAP		Activating	Lysosomal cyst(e)ine storage	[22]
PARK2	O60260	S127	ILHTDSRKDSP		Inhibitory	Prevents ubiquitinating activity	[23]
SCYL1	Q96KG9	S754	QPRPDSWGEDN		Modulatory	Golgi, endosome formation	[24]
SERBP1	Q8NC51	S25 S197,S199 S226,S234	LFDDESDPFEV EFDRHSGSDRSSF SHNWGTVKDELTESPKYIQ		Inhibitory	Translation reactivation	[25]
SNX16	P57768	S138,T146	KTPEESWVVFRRYTDSFRL		Inhibitory	Inhibition of autophagy	[26]
STK11IP	Q8N1F8	S404	RTLNPSPAGWF	FEVEL	Activating	Inhibition of autophagy	[27]
UNK	Q9C0B0	S255 S336 S359 S608,S611	HKYRSSPCPNV SSAVSSPTQPG VPVSPSSPHAP LGTSASHGSLGLNG		Inhibitory	Morphogenesis	[28]

^1^ HOXB13 phosphorylation in vitro was performed using immunoprecipitated mTOR complexes. However, cellular experiments with Torin or rapamycin yield similar observations making it likely that it is phosphorylated by mTORC1.

Three substrates, ATF4, LST2 and STK11IP, contain sequences corresponding to putative TOS (TOR signaling) motifs that are required for binding to the mTORC1-specific component Raptor and presentation to the catalytic mTOR sub-unit for phosphorylation [17,21,27]. The TOS consensus sequence is a five-amino-acid motif defined as F-X-Φ-(E/D)-Φ, where Φ represents a hydrophobic residue and X is any residue [29]. In all three cases, mutation of the highly conserved initial phenylalanine residue abolished Raptor binding/mTORC1 phosphorylation to confirm the functionality of these motifs. These examples add to the handful of known TOS-bearing mTORC1 substrates. Nevertheless, the absence of TOS-like sequences in the great majority of confirmed substrates begs the question as to how the majority of mTORC1 substrates are recruited.

In terms of relevance to cancer, phosphorylation of CDH1 at T129 transiently inactivates the APC/C^CDH1^ complex to promote glycolysis and cell cycle entry in normal cells [18]. Constitutive mTORC1 activation is a common event in many tumors, and this suggests an additional mechanism where mTOR could promote tumorigenesis by turning off cell cycle regulation by APC/C^CDH1^. Phosphorylation of the homeodomain-bearing transcription factor HOXB13 activates a pro-tumorigenic transcriptional program but at the same time, when phosphorylated, HOXB13 becomes targeted by the E3 ubiquitin ligase Skp2 for proteasomal degradation [20]. Battaglioni et al. [21] describe a crosstalk between the mTORC1 and EGFR pathways. Both pathways are frequently activated in cancer, with EGFR inducing an upstream signaling cascade to activate mTOR alongside other targets. mTORC1 phosphorylation stabilizes the EGFR negative regulator LST2 to dampen EGFR signaling and may potentially be anti-proliferative for EGFR-stimulated growth. Autoregulatory loops appear to be an important component of mTOR signaling. mTORC1 controls the level of mTORC2 activity by the phosphorylation of GRB10 and IRS1 (by S6K) to set up a negative feedback loop. mTORC2 in turn affects growth factor stimulation of mTORC1 by inhibiting TSC via Akt. mTORC1 also directly controls the localization and availability of TFEB and TFE3 that play a role in amino acid-dependent RAG activation of mTORC1.

### 4.2. mTORC2 Targets

Only three mTORC2 targets were reported within this time frame (Table 2). Surprisingly, one of the reported targets was the hydrophobic motif of S6K, a canonical mTORC1 substrate. To support this extraordinary claim, the authors assembled evidence from multiple lines of investigation such as TOS truncation S6K mutants, pharmacological inhibition using mTORC1-specific rapamycin vs. pan-mTOR active site inhibitors, shRNA knockdown of Raptor vs. Rictor, in vitro kinase assays using mTOR immunoprecipitated from cells undergoing various treatments, to make an interesting case for mTORC2 phosphorylation of S6K [30]. It should be noted that S6K phosphorylation by mTORC2 was previously reported but discounted as non-physiologically relevant [31]. Additionally, Rictor knockout cells show no reduction or loss of S6K phosphorylation. Nonetheless, S6K is anomalous among the AGC kinases in being phosphorylated by mTORC1 instead of mTORC2 and it is conceivable that under certain conditions, it could be subject to mTORC2-mediated phosphorylation.

**Table 2:** Direct substrates of mTORC2.

Substrate	Uniprot ID	Phosphorylated Site(s)	Phosphorylated Site(s) and Adjacent Residues	Purpose of Phosphorylation	Function	Reference
HSPB4	P02489	T148	GPKIQTGLDAT	Activating	Neuroprotection	[32]
PKN1	Q16512	S916	EAPTLSPPRDA	Activating, stability	Signaling	[33]
S6K1 ^1^	P23443-1	T412	VFLGFTYVAPS	Activating	Signaling, translation	[30]

^1^ refers to the 525 amino acid long α1 isoform with the hydrophobic motif at T412. The 502 amino acid long αII isoform (P23443-2, HM at T389) is more commonly referred to in the literature.

Adding to this debate, Taylor et al. have performed an extensive analysis of substrate selection and phosphorylation by mTORC2 [33]. Just as mTORC1 recruits some of its targets by a Raptor-binding TOS motif, they show that mTORC2 phosphorylation of the AGC kinases at the turn motif (TM) and hydrophobic motif (HM) is determined by selection at sites distal to the phosphosite. Substrates are recruited by mSin1, an mTORC2-unique component. Substrates bind to the CRIM (conserved region in the middle) domain of mSin1 for presentation to the mTOR active site. The mTORC2-Akt1 structure was resolved, and instead of local sequence elements adjacent to the phosphosite, the specificity determinants were secondary and tertiary structural elements of Akt that dictate binding to mSin1. Structural modeling of cryo-EM data identified a basic “N-lobe” region on Akt that interacts with the CRIM domain over a broad surface, including the previously described “acidic loop” that is necessary for Akt binding [34]. A second region, the C-lobe, was identified at the Akt kinase domain which binds the N-terminal regions of mSin1. Mutational analysis identified critical basic residues on the N-lobe (K158, K163, K182, K183 and R222), and corresponding alanine mutations severely impair Akt T450 and S473 phosphorylation. This mode of action is generalized for a related AGC kinase, PKN1, where phosphorylation of the S916 turn-motif is shown to be sensitive to mutation at the mSin1 acidic loop. Based on sequence conservation and structural considerations, they propose a common mechanism for mTORC2 phosphorylation of 18 AGC kinase family members (Akt1, Akt2, Akt3, SGK1, SGK2, SGK3, PKC_α_, PKC_β_, PKC_γ_, PKC_δ_, PKC_ε_, PKC_η_, PKC_θ_, PKC_ζ_, PKC_ι_, PKN1, PKN2, PKN3). Notable exceptions are the S6K kinases, which, as previously mentioned, are phosphorylated by mTORC1. S6K1 (P23443-2) shares sequence homology with Akt1 over the N-lobe, and the critical residues (K158, K162, K182, K183, Akt1 numbering) are conserved while R222 is substituted with the basic residue lysine in S6K1. It is possible that the S6K basic patch may have minimal functionality that can lead to occasional mTORC2 phosphorylation as has been reported. Nonetheless, it appears that the Raptor-binding TOS motif that is unique to S6K is dominant over the N-lobe basic patch, leading to preferential recruitment to Raptor and subsequent phosphorylation by mTORC1.

## 5. Conclusions

Experimental validation and target site identification of putative kinase targets are time-intensive and present a bottleneck in kinase studies. This is reflected in the modest number of novel targets identified in the period 2022–2025, despite mTOR signaling being a large and active field of research.

Only targets phosphorylated by mTORC1 and mTORC2 have been listed. However, there are reports of a non-canonical mTORC3 complex [35,36]. These complexes lack the defining Raptor and Rictor sub-units of mTORC1 and mTORC2 and have unique components instead (ETV7 or GIT1). No novel substrates are reported for either of these complexes, and there has been no independent confirmation of their existence. Nonetheless, they represent an intriguing possibility that may be worthy of investigation.

Despite steady progress in substrate identification, there remains a far greater number of unidentified targets than have been identified thus far. In the case of mTORC1, the overarching division of one of its roles as promoting anabolic processes and suppressing catabolism may offer a shortcut in identifying putative targets. For example, translation is the largest consumer of GTP [37]. GTP is converted from ATP by the nucleoside diphosphate kinases (NDPK). It is plausible that the high GTP demand during translation could be coordinated by mTORC1 through stimulating an increase in cellular GTP pools. NDPK is already known to be regulated by AMPK and histidine phosphorylation, and it would be tempting to see if mTORC1 similarly plays a regulatory role.

As a final note, HSPB4 was assigned in the publication [32] as an mTORC2 substrate on the basis of IP data but given that the kinase assay was performed using mTOR protein alone, which can indiscriminately phosphorylate both mTORC1 and mTORC2 substrates in vitro, its assignment as an mTORC2 target remains to be definitively confirmed.

## Data Availability

No new data were created or analyzed in this study.
